# Prioritizing the Timely Detection and Diagnosis of Early-Age Onset Cancer to Enable Optimal Disease Management and Outcomes

**DOI:** 10.3390/curroncol32070396

**Published:** 2025-07-10

**Authors:** Michael J. Raphael, Petra Wildgoose, Darren Brenner, Christine Brezden-Masley, Ronald Burkes, Robert C. Grant, Alexandra Pettit, Cassandra Macaulay, Monika Slovinec D’Angelo, Filomena Servidio-Italiano

**Affiliations:** 1 Sunnybrook Health Sciences Centre, Toronto, ON M4N 3M5, Canada; michaelj.raphael@sunnybrook.ca (M.J.R.); petra.wildgoose@sunnybrook.ca (P.W.); 2 Departments of Oncology and Community Health Sciences, Cumming School of Medicine, University of Calgary, Calgary, AB T2N 1N4, Canada; darren.brenner@ucalgary.ca; 3 Mount Sinai Hospital, Toronto, ON M5G 1X5, Canada; christine.brezden@sinaihealth.ca (C.B.-M.); ron.burkes@sinaihealth.ca (R.B.); 4 Princess Margaret Cancer Centre, University Health Network, Toronto, ON M5G 2C4, Canada; robert.grant@uhn.ca; 5 Horizon Health Network, Fredericton, NB E3B 3B7, Canada; dr.alexandra.pettit@horizonnb.ca; 6 Colorectal Cancer Resource & Action Network (CCRAN), Toronto, ON M4W 3E2, Canada; cassandra.m@ccran.org; 7 School of Psychology, University of Ottawa, Ottawa, ON K1N 6N5, Canada; mslovin2@uottawa.ca

**Keywords:** early-age onset cancer, EAOC, disease awareness, comprehensive genomic profiling

## Abstract

In November 2024, the fourth annual Symposium focusing on early-age onset cancer (EAOC) was hosted by the Colorectal Cancer Resource & Action Network (CCRAN), assembling clinicians, researchers, and patients virtually to discuss challenges in early detection and diagnosis of individuals afflicted with EAOC across tumour types. The meeting addressed the rising rates of EAOC and identified strategies to overcome barriers to timely detection and diagnosis by closing gaps in public and healthcare provider knowledge on symptoms of cancer in younger adults and reducing inequities in standard screening for younger age groups. Discussions also encompassed the various factors that serve as impediments to accessing diagnostic testing and obtaining results, as well as the critical need for access to diagnostics such as comprehensive genomic profiling (CGP), the results of which could be imperative in helping to guide clinical decisions regarding effective and well-tolerated targeted therapies. The Symposium generated key calls to action regarding increasing EAOC education and awareness among primary care providers and the public, re-evaluation of cancer screening programs’ eligibility criteria to include younger populations, and mechanisms to reduce waiting times for diagnostic testing by addressing technologist shortages and improving access to CGP through national collaborative strategies and increased funding.

## Simple Summary

In 2024, the Colorectal Cancer Resource & Action Network hosted a virtual Symposium to discuss barriers to early detection and diagnosis of individuals diagnosed with early-age onset cancer across tumour types. Clinicians, researchers and patients assembled to generate strategies to overcome challenges to the early detection and diagnosis of early-age onset cancer, and to facilitate timely access to diagnostic testing and results in order to help inform treatment decisions that optimize quality of life and longevity. Over the course of the Symposium, several key themes were identified for collaborative action which included: increasing education and awareness of early-age onset cancer among both primary care providers and the public, re-evaluation of the eligibility criteria for cancer screening programs to include younger populations, and strategies and solutions to reduce waiting times for diagnostic testing by addressing technologist shortages and improving access to comprehensive genomic profiling through national collaborative strategies with increased funding.

## 1. Introduction

In recent decades, a rising incidence of early-age onset cancer (EAOC, defined as cancers in adults under the age of 50 years old) has been detected in various regions of the world, impacting at least 44 developed countries, including Canada [1,2,3]. The exact etiology of this increase is yet to be firmly established. Multiple hypotheses have been put forward, including dietary, lifestyle, environmental, and metabolic factors resulting in changes to the gut microbiome and creating a pro-carcinogenic state [4,5,6]. The implications are particularly detrimental given that those under 50 years old have unique physical and psychological concerns that can be adversely affected by both disease and treatment [7,8,9]. In addition, they are of working age and often have young families to support. Early detection and diagnosis are not only critical to initiating the patient’s care pathway, but have been demonstrated to lead to less advanced disease and improved patient outcomes.

In 2023, the Colorectal Cancer Resource & Action Network (CCRAN) expanded its annual symposia series to include all cancers affecting young adults [10], and the 2024 Symposium built on this work by providing an opportunity for patients with EAOC, caregivers, patient advocacy groups, oncology professionals, pathologists, primary care providers (PCPs), researchers, policy-makers, and industry professionals to share outputs and progress made in the past year (Appendix A, Appendix B and Appendix C). A central feature of the 2024 Symposium was the presentation of key findings from CCRAN’s National Pan-Tumour Patient Survey which examined priorities, challenges, and experiences of individuals with EAOC.

The goal of the 2024 Symposium was to advance the discussion on actionable strategies and recommendations for facilitating early detection and timely access to diagnostic testing for those at risk for EAOC. The Symposium agenda (Appendix D) was designed to stimulate discussions around healthcare barriers commonly experienced by patients, explore recent progress, and determine concrete steps forward.

## 2. Increasing Early Detection and Diagnosis of EAOC

Throughout the Symposium, patient speakers affected by different cancer types shared their healthcare experiences and corroborated the concept of delayed diagnosis as a hallmark of EAOC. Discussions focused on two major barriers to the diagnosis of EAOC: low PCP/public awareness of EAOC and gaps in screening eligibility for younger age groups across the country.

### 2.1. Barrier to Early Detection: Lack of Awareness of EAOC

Awareness campaigns to familiarize the public with the symptoms that are consistent with the various cancer types have had some success [11,12,13], but there remains a detrimental lack of awareness of cancer risk factors, signs, and symptoms among younger Canadians and PCPs that ultimately poses a major barrier to timely EAOC detection. Symposium patient panelists described a singular experience when seeking a diagnosis for initial symptoms of cancer, reporting that their PCP misdiagnosed them with a more minor condition, deeming them to be at minimal risk of cancer because of their young age and otherwise healthy status. These patient narratives were substantiated by CCRAN’s 2024 National Pan-Tumour Patient Survey findings, which represented a collaboration of 15 cancer patient organizations. The survey’s objective was to understand patient priorities and barriers to detection and diagnosis for individuals in Canada who were diagnosed with cancer between 18 and 49 years of age in the last ten years.

• One hundred and seven respondents completed the survey, with representation from all 10 Canadian provinces.
• Respondents reported diagnosis of 10 tumour types (primarily colorectal and neuroendocrine); 42% were diagnosed at Stage IV.
• Eighty-one percent reported that their cancer was self-detected through self-screening or the presence of symptoms.
• Only 2% of respondents had their EAOC first detected through routine checkups with their family doctor, suggestive of missed opportunities in primary care.
• Self-dismissal was prevalent: 42% of respondents waited > 5 weeks to schedule an appointment.
• Two-thirds of respondents felt that their provider minimized their symptoms, which were perceived as due to their young age (78%) and vague symptoms (41%).
• One-third of respondents reported first obtaining an accurate diagnosis of EAOC in an emergency room (ER).
• Thirty-four percent of patients were diagnosed with EAOC in hospital, while only twelve percent of patients received their diagnosis from their PCP.
• Thirty-five percent of respondents reported that it was difficult/very difficult to access care for diagnosis.

The survey findings corroborate recent Canadian and international research demonstrating that when young patients do present to their healthcare provider (HCP) with cancer symptoms, their concerns are not being validated or adequately evaluated [14,15,16,17]. However, it is important to note that PCPs are in the challenging position of providing optimal patient care and determining diagnoses for oftentimes common or vague symptoms that have a multitude of possible causes, while working within a struggling healthcare system that has significant diagnostic delays. Previous research suggests that an ER diagnosis of EAOC is indicative of a common and frustrating journey in which patients are unable to access sufficient care—primarily due to misdiagnoses or being among the 20% of Canadians without a PCP [18]—until their symptoms worsen to a severity where an ER visit becomes necessary [19,20]. This leads to a cancer diagnosis in an unsuitable and hectic environment, an experience described by patients as distressing [21], further highlighting the failure of healthcare systems to scaffold adequate primary care-initiated pathways for the diagnosis of suspected EAOC. Unsurprisingly, the 2024 survey results found that the most common unmet need for newly diagnosed patients was timeliness of detection, emphasizing the importance of increasing awareness of EAOC in both the public and HCP spheres.

#### 2.1.1. Recent Progress at Increasing EAOC Awareness

Patient advocacy groups have enacted strong recent efforts to increase public awareness of EAOC and engage with local communities to disseminate relevant information and reduce stigma associated with many tumour types. Groups including The Walnut Foundation, Lung Cancer Canada, and the Canadian Breast Cancer Network have developed tools and programs, including information sessions featuring medical experts to connect with the public and challenge myths that cancer is exclusively a disease of old age. To educate the Canadian public regarding the early signs and symptoms of colorectal cancer (CRC), promote screening in the eligible population, and raise awareness of the rising incidence and prevalence of this disease in adults of 50 years of age and younger, CCRAN has created the Jumbo Colon exhibit, a large-scale inflatable walk-through replica of a colon; since 2023, the Jumbo Colon has been featured in 30 locations, and attended by approximately 5200 visitors. CCRAN has also developed a CRC awareness toolkit which includes preventative tips and a list of questions to ask PCPs about screening.

Initiatives to raise EAOC awareness have extended beyond those directed at the public, with patient advocacy groups recognizing that PCPs are in the privileged position to investigate symptoms consistent with EAOC regardless of the patient’s age. To assist populations that are disproportionately affected by certain cancers, Queering Cancer provides training and resources for HCPs of sexually and gender-diverse communities. Additionally, health organizations, such as Cancer Care Ontario (CCO), have created virtual resources targeting low EAOC awareness among PCPs [22] to educate them on presenting symptoms for different tumour types prevalent among young adults and ultimately allow faster detection of cancer.

#### 2.1.2. Building on Momentum

In order to build on the recent work prioritizing the detection of EAOC, further education and awareness campaigns are needed to increase the index of suspicion of cancer among PCPs and to inform younger Canadians about the risk of developing cancer in their age group so they are best able to advocate for themselves. CCRAN has developed a hub on their website designed specifically for Canadians under 50 to receive more education and information on EAOCRC. Additionally, CCRAN has embarked on a Jumbo Colon tour, promoting awareness of EAOCRC as they bring this interactive exhibit to Canadian communities, including Indigenous populations.

### 2.2. Barrier to Early Detection: Lack of Sub-Optimal up Take of Screening Programs

Cancer screening has been proven to be a successful tool in reducing cancer incidence rates and mortality [23]. Provincial screening programs across Canada for breast cancer and CRC have resulted in a significant decrease in the burden of cancer for these tumour types [24,25]. However, the value and impact of screening programs rely on their eligibility criteria, and current programs for many tumour types, including prostate and colorectal, employ age- and family-history-related restrictions derived from out-dated evidence regarding populations at risk, with younger adults typically excluded [26,27]. Additionally, for tumour types including colorectal, prostate, breast, and gastroesophageal, there are pervasive public perceptions that younger populations are not at risk, presenting an additional barrier to screening and detection [28,29,30,31]. This is further exacerbated by prevalent stigmas regarding the etiology of some tumours (i.e., those afflicted must have engaged in risky behaviours) or misconceptions regarding cancer being strictly an older person’s disease [32,33,34,35,36,37,38], and it is unsurprising that only one-quarter of respondents to the 2024 National Pan-Tumour Patient Survey reported that they prioritized understanding the cause of their cancer. Finally, inequities in access to screening are evident, with individuals considered to be socio-economically disadvantaged having lower rates of screening than those with higher incomes [39,40,41].

While screening rates are sub-optimal for many provincial cancer detection programs, population-based screening programs do not even exist for other tumour types, including gastroesophageal, pancreatic, skin, and cholangiocarcinoma/hepatocellular cancers. Due to a lack of standardized detection approaches, these cancers are typically diagnosed either by incidental finding or only after the tumour has progressed to a symptomatic stage. Population-based screening remains a critical unmet need.

#### 2.2.1. Recent Approaches to Reduce Inequities in Cancer Screening

In Canada, cancer screening programs are typically conducted as provincial initiatives or as a combination of provincial programs in conjunction with PCPs. As a result of updated epidemiological research demonstrating the risk of breast cancer in young women, and in recognition of the need for early detection to access timely treatment, the minimum age criterion for government-funded breast cancer screening programs was lowered from 50 to 40 years in Ontario and Newfoundland in 2024 [42,43]. Similar eligibility modifications have been implemented or are currently being applied through a phased approach in other regions, including British Columbia, Nova Scotia, Prince Edward Island, Saskatchewan, and Manitoba [44,45,46,47,48].

To address existing gaps in EAOC detection, programs have recently been developed by PCPs and patient groups. The Walnut Foundation has partnered with HCPs to reach the Black male community and overcome common barriers to access by conducting free prostate-specific antigen (PSA) blood screening at community pop-up clinics across Ontario, a province where screening is not currently governmentally funded for asymptomatic individuals [49]. In 2024, British Columbia became the first Canadian province or territory to offer self-screening for human papillomavirus (HPV), the leading cause of cervical cancer, through at-home kits, thereby facilitating public access and convenience, particularly for underserved and disadvantaged populations [50].

#### 2.2.2. Building on Momentum

To adequately respond to the increase in EAOC, screening programs must be modified to include younger populations. For example, current screening guidelines for CRC recommend testing asymptomatic, average-risk individuals between 50 and 74 years, ignoring the epidemiological evidence demonstrating that these age restrictions must be lowered to 45 years to more optimally detect disease in younger adults [27].

Additionally, further research is needed to inform the development of targeted screening programs to most effectively capture at-risk individuals. There is a dearth of supportive research data for non-traditional risk factors for EAOC, information that is imperative for the development of evidence-based updates to risk factor models. Research to explore the genomic basis for early-onset risk is also needed, as this knowledge can be leveraged into more refined approaches for risk-stratified screening strategies that are cost-effective and can support early detection.

## 3. Improving Timely and Equitable Access to Diagnostic Testing

Timely diagnostics are needed for optimal patient outcomes, but gaps currently exist in providing patients with access to both diagnostic testing and their results, delaying treatment further. Evidence suggests that a late-stage diagnosis does not completely account for the aggressiveness of EAOC, translating to an even stronger need for more timely access to treatment. Symposium panelists described various barriers that must be quickly and fully addressed to improve access to diagnostic testing and results.

### 3.1. Barrier to Accessing Diagnostic Testing

Diagnostic imaging and laboratory medicine commonly serve as the gateway to chemotherapy, surgery, and other follow-up plans, but despite this essential role, the field is plagued with multiple barriers that limit patient access to testing, delay treatment, and cause devastating consequences to the mental health of patients with EAOC. The primary obstacle is staff shortages [51]: of 226 accredited laboratories in Ontario, 83% are currently experiencing medical laboratory technologist (MLT) shortages, which is projected to worsen as 42% of MLTs meet retirement eligibility over the next few years. CCO has established a 14-day turnaround time standard for surgical pathology results in Ontario, which is not consistently met by the majority of laboratories [52], two-thirds of which attribute the shortfall to lack of MLTs. Waiting times for CT scans and magnetic resonance imaging (MRI) far exceed all standards across many provinces and territories [53], and once tests are completed, there can be additional delays in having the results interpreted, creating a very difficult situation for patients with EAOC. While the expansion of cancer screening eligibility criteria is necessary for early detection, the capacity of the healthcare system to adapt to increased testing needs has been sub-optimal and must be addressed.

#### 3.1.1. Recent Progress

Several initiatives are currently underway to target the significant barriers to accessing diagnostic testing and obtaining results in a timely manner. In 2023, the government of Ontario granted free tuition to students who enrolled in MLT training programs at specific colleges, to promote workforce growth in regions of the province with high-priority and underserved communities [54]. During the following year, the province expanded undergraduate programs to include an additional 700 seats for MLTs and medical radiation technologists (MRTs) [55]. Additional requests have been directed to the government for more clinical placement funding for MLTs as well as for the development of simulation training laboratories across Ontario. CCRAN has also been working to highlight the MLT shortage in Ontario through awareness campaigns, calling on the provincial and territorial governments to improve MLT retention strategies, and invest in rural and remote areas where shortages are particularly evident [56].

#### 3.1.2. Building on Momentum

In order to have the greatest chance of successfully reducing diagnostic delays, patient advocacy groups and HCPs should continue to collaborate with provincial associations for imaging and laboratory technologists when lobbying health policy-makers. These groups must present a unified voice to educate the public and the provincial government on where the severe gaps are in current cancer diagnostic workflows, highlight staffing shortages and the distressing consequences, and identify where funding is needed to increase equitable access to medical imaging and diagnostics across the country.

### 3.2. Barriers to Accessing Comprehensive Genomic Profiling (CGP)

Understanding treatment options is a key priority for individuals diagnosed with cancer, as reported by 98% of the 2024 National Pan-Tumour Survey respondents. Comprehensive genomic profiling (CGP), a next-generation sequencing technology that analyzes a patient’s cancer genes simultaneously to identify genomic alterations or mutations, is invaluable in guiding clinical decisions of novel, targeted therapies that have biological rationale, as well as the order of therapeutic interventions [57,58]. When oncologists have CGP results early on in the cancer care journey for patients with EAOC, they can determine whether the patient is a candidate for precision medicine that may be effective and well-tolerated. Access to precise treatment options with fewer side effects is particularly relevant for younger patient populations that are in the midst of their careers and may also have families to support. Additionally, the results of CGP can identify actionable mutations that can serve as an entry to clinical trial access or off-label therapy use. However, significant barriers to CGP access exist. Testing is not consistently funded across all provinces and territories, resulting in geography-based inequities. This is especially devastating given that individuals with EAOC rarely meet the age-related eligibility criteria for program or grant funding for cancer testing. Moreover, only large urban centres typically have the technology for CGP while rural areas do not have these resources, creating more disparities in access.

Based on the 2024 survey results, a severe knowledge gap exists regarding CGP: only one-third of respondents reported having discussions with their cancer care team regarding testing, despite 56% having been diagnosed with metastatic cancer. CGP was not offered to two-thirds of respondents, and more than half reported that they would like to access testing to determine whether additional treatment options exist. Only one-third of respondents with metastatic cancer accessed CGP, while an additional 32% of respondents who wanted the testing were unable to receive it, indicative of a very significant care gap. Given that CGP is not commonly publicly funded, it is reasonable to assume that the tremendous financial burden of testing is contributing to the inequity in access for individuals with EAOC.

#### 3.2.1. Recent Progress

In 2024, a Canadian mixed-methods study of 55 adults diagnosed with CRC was conducted to explore patient experiences with biomarker testing [59]. Of the one-third of respondents that were not tested, the primary reason was that it was not offered, representing a significant gap. Following the 2024 Symposium, CCRAN initiated a partnership to conduct a cost–benefit analysis of CGP across five types of metastatic cancer in Canada in order to move the needle forward on access to CGP. The findings will provide much-needed national data to help inform funding decisions regarding CGP becoming a standard of care in Canada, and will highlight the impact not only on patient survival, but also on their quality of life and longevity, as well as on the healthcare system by identifying the treatment options that may improve patient outcomes and reduce the need for lengthy, complicated, and costly services that are less likely to provide benefit.

#### 3.2.2. Building on Momentum

CGP is the strongest existing tool to help patients understand their cancers, identify optimal therapeutic options, and participate in informed, shared decision-making about the treatment plan that may provide the greatest chance of an optimal outcome. The next steps in breaking barriers around access to CGP is to ensure all oncologists are engaging patients in discussions about testing at diagnosis, and that provincial and territorial governments are reducing inequities in access to these crucial tests.

## 4. Conclusions

CCRAN’s two-day virtual Symposium amassed experts from Canada and around the world to discuss how to prioritize early detection and diagnosis of EAOC and facilitate timely access to diagnostic testing and results to help inform optimal treatment decisions. To continue the collaborative engagement, discuss recent progress in advancement towards these goals, and further investigate the themes that arose from the meeting, a fifth Symposium has been planned for November 2025. Primary efforts will focus on methods to transform healthcare systems in order to support EAOC detection, diagnosis, and care and to discuss how progress has ensued through collaborative action generated by the 2024 Symposium, which prioritized the timely detection and diagnosis of EAOC as a catalyst to optimal disease management and treatment.

The Symposium generated the following key calls to action:

1. EAOC education for PCPs is needed to ensure that, as primary access points to cancer detection, these providers consider cancer as a potential diagnosis in all patients presenting with relevant symptoms. Educational initiatives should include medical school training and continuing medical education (CME) to improve recognition of EAOC signs and symptoms, as well as resources and toolkits created by patient advocacy groups for convenient reference during patient visits.
2. To facilitate self-advocacy in young adults, funding must be allocated for government campaigns to implement EAOC promotions aimed at increasing public awareness, as well as for patient advocacy group engagement at the community level to educate the public on EAOC in their age group while dispelling misconceptions regarding populations at risk.
3. The eligibility criteria of cancer screening programs across Canada should be re-evaluated to capture a younger population, with funding allocated to amend existing programs, including the lowering of eligibility age for average-risk colorectal cancer screening to 45 years. Funding should also be assigned to develop new programs to protect all Canadians from the onset of cancer, such as accessible mole mapping for all those with a family history of skin cancer or who are considered high-risk, and H. pylori testing for those with related GI symptoms or a history of peptic ulcer disease, with the ultimate goal of effectively promoting these screening initiatives.
4. As standard of practice, oncologists should routinely engage all patients with EAOC in discussions about CGP so that patients are well-informed regarding the available tools to help them understand their tumour and their treatment options.
5. Investment should be made towards research to establish evidence-based approaches to identifying populations at risk for EAOC, through determination of risk factors for various tumour types, to inform targeted screening programs.
6. To expedite diagnosis of EAOC, equitable access to medical imaging is needed for all Canadians through mechanisms including federal and provincial funding for more radiology equipment, an increased number of both technologist education programs and students accepted, fair compensation of technologists to incentivize them to remain in the healthcare system, pan-Canadian licensure for MRTs, and a framework for international radiology professionals to navigate the Canadian system and enter the workforce safely.
7. Investment is needed to expedite rapidly available and accessible laboratory testing and results to inform treatment decisions for Canadians with EAOC. Additionally, funding to increase MLT clinical placements is needed to provide students with crucial practical experience in accredited laboratories, allowing them to develop the skills and knowledge required to successfully enter the workforce and shorten current turnaround times for testing results.
8. Facilitation of collaborations between regulators, payers, and industry is necessary to create a national strategy for equitable access to CGP for all individuals with EAOC. Public funding of CGP by all provinces and territories will be critical in reducing the significant financial burden of cancer incurred by patients at a time when working may not be an option, so that all individuals diagnosed with metastatic EAOC can have the best opportunity to identify tailored treatment options with optimal risk/benefit profiles.

## Data Availability

Data presented at the Symposium are included in this article. No further data sharing is applicable.

## Appendix A. Symposium Organization

The 2024 Symposium was organized by CCRAN, a national patient advocacy group which provides CRC patient and caregiver support, education, and advocacy in Canada. CCRAN has recently expanded its patient-focused mandate across multiple tumour types, as reflected in the Symposium content.

This virtual two-day meeting was overseen by an Expert Steering Committee that provided direction on the objectives, agenda, and speakers. The committee members were:

• Dr. Michael Raphael (Odette Cancer Centre, Sunnybrook Health Sciences Centre, Toronto, ON, Canada)—Co-Chair;
• Dr. Petra Wildgoose (Young Adult Colorectal Cancer Program, Sunnybrook Health Sciences Centre, Toronto, ON, Canada)—Co-Chair;
• Dr. Darren Larsen (Cancer Quality Council of Ontario, Toronto, ON, Canada);
• Dr. Catalina Lopez-Correa (Genome Canada, Ottawa, ON, Canada);
• Mr. Christopher Mammoliti (EAOC Patient Expert; Thyroid Cancer Survivor and Stage 4 Colon Cancer Survivor);
• Dr. Mita Manna (Saskatchewan Cancer Centre, Saskatoon, SK, Canada);
• Dr. Alexandra Pettit (Horizon Health Network, Fredericton, NB, Canada).

## Appendix B. Collaborating Patient Advocacy Groups and Partners

**Table A1:** Collaborating patient advocacy groups and partners.

Patient Advocacy Groups	
All.Can Canada	Lung Health Foundation
AYA CAN	Lymphoma Canada
Canadian Breast Cancer Network	My Gut Feeling
Canadian Cancer Survivor Network	Myeloma Canada
Canadian Neuroendocrine Tumour Society	Queering Cancer
Canadian Organization for Rare Disorders	Pancreatic Cancer Canada
Cholangio-Hepatocellular Carcinoma Canada	Pink Pearl
Craig’s Cause Pancreatic Cancer Society	Praxus Health
Dense Breasts Canada	Prevent Cancer Now
Genomic Focus	Prostate Cancer Foundation Canada
GIST Sarcoma Life Raft Group Canada	Rethink Breast Cancer
Leukemia & Lymphoma Society of Canada	Save Your Skin Foundation
Lung Cancer Canada	The Walnut Foundation
**Industry Partners**	
AstraZeneca	Merck
GSK	Takeda
Amgen	Bayer
Coulson Contracting Ltd.	Incyte
Johnson & Johnson	Signatera
**Media Partner**	
GlobalNews	

## Appendix C. Symposium Organization

The Symposium was attended by 455 participants, representing various stakeholder groups, specifically healthcare professionals, patients, caregivers, industry partners, researchers, and policy-makers. Registrants were from Canada, the United States, France, Germany, Great Britain, India, Mexico, Australia, Brazil, Indonesia, Austria, Hong Kong, Italy, Japan, Malta, Peru, Portugal, Russia, Saudi Arabia, Spain, Switzerland, and Taiwan.

## Appendix D. Symposium Agenda

The meeting agenda is presented in Table A2. All sessions were held virtually.

**Table A2:** Symposium agenda.

Session	Speakers
**Day 1:** Priorities for early-age onset cancer detection and diagnosis: drawing government attention to the importance of timely diagnosis and reducing screening age ***Moderator******:*** **Dr. Monika Slovinec D’Angelo,** Chief Research Officer, CCRAN	
Symposium opening	**Filomena Servidio-Italiano,** President and CEO, CCRAN **Christopher Mammoliti,** EAOC Patient Expert; Thyroid Cancer Survivor and Stage 4 Colon Cancer Survivor
Key learnings from CCRAN’s 2023 EAOC Symposium	**Dr. Michael Raphael,** Medical Oncologist, Odette Cancer Center, Sunnybrook Health Sciences Centre **Dr. Petra Wildgoose,** Primary Care Physician and Lead, Young Adult Colorectal Cancer Clinic, Odette Cancer Center, Sunnybrook Health Sciences Centre
Understanding EAOC patient priorities and barriers to detection and diagnosis: survey findings	**Dr. Monika Slovinec D’Angelo,** Chief Research Officer, CCRAN **Cassandra Macaulay,** Deputy Chief Research Officer, CCRAN **Filomena Servidio-Italiano,** President and CEO, CCRAN
Understanding the increased prevalence and underlying factors of EAOC	***Moderator:*** **Christopher Mammoliti,** EAOC Patient Expert; Thyroid Cancer Survivor and Stage 4 Colon Cancer Survivor ***Panelists:*** **Jason Abramovitch,** Early-Age Onset Metastatic Colorectal Cancer Survivor **Dr. Darren Brenner,** Associate Professor, Departments of Oncology and Community Health Sciences, Cumming School of Medicine, University of Calgary **Dr. Meg Sears,** Chair, Prevent Cancer Now **Dr. Tomotaka Ugai,** Faculty Member, Cancer Epidemiology Program, Dana-Farber/Harvard Cancer Center
Recommendations for EAOC screening programs: opportunities for collaborative advocacy efforts	***Moderator:*** **Jenn Gordon,** Lead, Strategic Operations and Engagement, Rethink Breast Cancer ***Panelists:*** **Teresa Tiano,** Chair and Co-Founder, My Gut Feeling; Stomach Cancer Survivor and a 9-Time Cancer Survivor **Cassandra Macaulay,** Deputy Chief Research Officer, CCRAN **Michele Wright,** Coordinator, Patient Care Initiatives, Lung Cancer Canada **Ken Noel,** Executive Director, The Walnut Foundation; Black Prostate Cancer Survivor **Kathleen Barnard,** Founder and President, Save Your Skin Foundation (SYSF) **Brenda Clayton,** President and Founder, Cholangio-Hepatocellular Carcinoma Canada; Caregiver of Daughter who succumbed to Early-Age Onset Cholangiocarcinoma
Prioritizing timely and equal access to diagnostic testing in cancer care: examining policy options	***Moderator:*** **Dr. Monika Slovinec D’Angelo,** Chief Research Officer, CCRAN ***Panelists:*** **Sam Karikas,** Invasive Ductal Cell Carcinoma Patient **Dr. Darren Larsen,** Chair, Cancer Quality Council of Ontario **Dr. Ania Kielar,** President, Canadian Association of Radiologists **Michelle Hoad,** CEO, Medical Laboratory Professionals’ Association of Ontario (MLPAO) **Dr. Jason Karamchandani,** Associate Professor, Departments of Pathology, Neurology and Neurosurgery, McGill University
Using technology to expand cancer screening	**Dr. Iris Gorfinkel,** Principal Investigator and Founder, PrimeHealth Clinical Research
Advocating for prompt detection and diagnosis of early-age onset cancer	***Moderator:*** **Laura Greer,** Senior Vice President and National Health Sector Lead, Health and Wellness, Hill & Knowlton; Breast Cancer Advocate ***Panelists:*** **Christopher Mammoliti,** EAOC Patient Expert; Thyroid Cancer Survivor and Stage 4 Colon Cancer Survivor **Joanne Nagy,** Stage 2 Invasive Ductal Carcinoma Patient, Triple Positive **Katie Hulan,** Lung Health Advocate; Early-Age Onset Stage 4 ALK Positive Lung Cancer Patient **Thomas Flannery,** Stage 4 Prostate Cancer Patient; Prostate Cancer Foundation Canada **Elise Gasbarrino,** Founder and Executive Director, Pink Pearl Canada; Early-Age Onset Ovarian Cancer **Dr. Neety Panu,** Lead Radiologist, Sioux Lookout Meno Ya Win Health Centre
**Day 2: The unique needs and optimal management for early-age onset cancer (EAOC) patients** ***Moderator:*****Dr. Monika Slovinec D’Angelo,** Chief Research Officer, CCRAN	
Welcome from CCRAN’s President	**Filomena Servidio-Italiano,** President and CEO, CCRAN **Eric Hamilton,** Stage 4 Colorectal Cancer Patient
Optimizing care for early-age onset cancer patients: the role of specialized clinics	***Moderator:*** **Dr. Usmaan Hameed,** Colorectal Surgical Oncologist, North York General Hospital ***Caregiver:*** **Stephanie Florian,** Weather Anchor, Reporter and Actor; Caregiver of spouse who succumbed to early-age onset metastatic colorectal cancer ***Panelists:*** **Jennifer Catsburg,** Clinical Nurse Specialist, Adolescent and Young Adult Program, Princess Margaret Cancer Centre, University Health Network **Dr. Karen Fergus,** Clinical Psychologist, Odette Cancer Center, Sunnybrook Health Sciences Centre **Bridget Veltri,** Certified Child Life Specialist, Hamilton Health Sciences—McMaster Children’s Hospital and Juravinski Hospital and Cancer Centre **Dr. Usmaan Hameed,** Colorectal Surgical Oncologist, North York General Hospital
The role of psychosocial support for young adults living with cancer and their families	***Moderator:*** **Cassandra Macaulay,** Deputy Chief Research Officer, CCRAN ***Panelists:*** **Julia Girmenia,** Key Collaborator, Rethink Breast Cancer; Stage 4 Early-Age Onset Inflammatory Breast Cancer Patient **Kathryn Hum,** Patient Advocate; Early-Age Onset De Novo Metastatic Breast Cancer Patient **Anwar Knight,** Award-Winning Broadcaster, Producer and Environmentalist; Hodgkin’s Lymphoma Survivor **Dr. Mary Jane Esplen,** Professor, Department of Psychiatry, Temerty Faculty of Medicine, University of Toronto **Dr. Sasha Mallya,** Clinical Psychologist, Department of Psychosocial Oncology, Tom Baker Cancer Centre **Rachelle Kosokowsky,** Clinical Oncology Social Worker, Saskatchewan Cancer Agency **Dr. Karine Bilodeau,** Associate Professor, Faculty of Nursing, University of Montreal
The role of the gut microbiome in cancer treatment	**Dr. Arielle Elkrief,** Assistant Professor, Dept. of Hemato-Oncology, the University of Montreal Hospital Research Centre
What’s new in the management of metastatic cancer?	***Moderator:*** **Dr. Joseph C. Del Paggio,** Medical Oncologist and Chief of the Department of Oncology, Thunder Bay Regional Health Sciences Centre-Regional Cancer Centre ***Panelists:*** **Dr. Christine Brezden-Masley,** Medical Oncologist and Assistant Professor, Chair of the HPB Cancer Disease Site Group Halifax, Division of Medical Oncology and Department of Community Health and Epidemiology, Nova Scotia Cancer Centre and Dalhousie University **Dr. Ravi Ramjeesingh,** Medical Oncologist and Assistant Professor, Chair of the HPB Cancer Disease Site Group Halifax, Division of Medical Oncology and Department of Community Health and Epidemiology, Nova Scotia Cancer Centre and Dalhousie University **Dr. Helen MacKay,** Medical Oncologist, Odette Cancer Center, Sunnybrook Health Sciences Centre **Dr. Ronald Burkes,** Medical Oncologist, Mount Sinai Hospital/Princess Margaret Cancer Centre/University Health Network **Dr. Pashtoon M. Kasi,** Medical Director, GI Medical Oncology, City of Hope **Dr. Jose Perea,** Chief, Department of Surgery, Vithas Arturo Soria University Hospital
Streamlining younger cancer patients’ clinical care pathways: the value of advanced biomarker testing	***Moderator:*** **Dr. Cathy Eng,** Vanderbilt-Ingram Cancer Center ***Patient:*** **Suzanne Wood,** Early-Age Onset Stage 4 Colon Cancer Patient ***Panelists:*** **Dr. Shaqil Kassam**, Medical Oncologist, Stronach Regional Cancer Centre **Dr. Shantanu Banerji,** Medical Oncologist, CancerCare Manitoba **Dr. Monika Slovinec D’Angelo,** Chief Research Officer, CCRAN **Dr. Robert Grant,** Medical Oncologist, Princess Margaret Cancer Centre, University Health Network
Cancer Patient Empowerment Program (Cancer PEP) *featured video*	**Dr. Rob Rutledge,** Co-Founder, CancerPEP.org; Radiation Oncologist; Associate Professor, Dalhousie University, Halifax, Nova Scotia **Dr. Gabriela Ilie,** Co-Founder, CancerPEP.org; Associate Professor of Epidemiology, Faculty of Medicine, Dalhousie University
Unlocking the potential of your tumour’s biomarker status: a novel tool to identify targeted treatments and clinical trials	**Matt Reidy,** Founder, Genomic Focus; Long-term, Stage 4 Cancer Survivor
The importance of information sharing and the role of patient groups in promoting health equity	***Moderator:*** **Michelle Audoin,** Patient Advocate and Community Collaborator; All.Can Canada Evidence Working Group ***Panelists:*** **Dr. Amanda Bolderston**, Co-founder, Queering Cancer **Anthony Henry**, President, The Walnut Foundation **Jenn Gordon**, Lead, Strategic Operations and Engagement, Rethink Breast Cancer **Bukun Adegbembo**, Director of Operations, Canadian Breast Cancer Network **Frank Pitman**, Outreach and Volunteer Coordinator, CCRAN **Michele Wright**, Coordinator, Patient Care Initiatives, Lung Cancer Canada **Madison Fullerton**, Vice President, Operations & Community Partnerships, Praxus Health
Closing remarks	**Filomena Servidio-Italiano,** President and CEO, CCRAN
